# Trends in the ease of cigarette purchase among Korean adolescents: evidence from the Korea youth risk behavior web-based survey 2005–2016

**DOI:** 10.1186/s12889-018-6151-9

**Published:** 2018-11-07

**Authors:** Seo Young Kang, Jung Ah. Lee, Hong-Jun Cho

**Affiliations:** Department of Family Medicine, Asan Medical Center, University of Ulsan College of Medicine, 88, Olympic-ro-43-gil, Songpa-gu, Seoul, 05505 South Korea

**Keywords:** Ease of cigarette purchase, Korean adolescents, Youth, Smoking

## Abstract

**Background:**

According to the Juvenile Protection Act in Korea, no one is allowed to sell, rent, or distribute tobacco products to adolescents. Furthermore, the Framework Convention on Tobacco Control Article 16 prohibits the sale of tobacco products to minors. In this study, we investigated the trends in and associated factors of the ease of cigarette purchase among Korean adolescents from 2005 to 2016.

**Methods:**

The analyses were based on the data of the Korea Youth Risk Behavior Web-based Survey. We estimated the trends in the ease of cigarette purchase from 2005 to 2016 and evaluated associated factors. Ease of cigarette purchase was defined as the proportion of adolescents who were able to purchase cigarettes from among those who had tried to purchase cigarettes in the past 30 days.

**Results:**

The ease of cigarette purchase began decreasing since 2008 (81.3%) compared to 2005 (83.9%). It decreased to 76.5% in 2013 and further decreased to 71.4% in 2016. The ease of cigarette purchase increased for adolescents who were in higher grades, smoked larger amounts of cigarettes per day, lived in metropolitan cities, had problematic drinking in the past year, and had close friends who smoked. It decreased in adolescents who had current smokers among their family members.

**Conclusions:**

Although the proportion of adolescents who were able to purchase cigarettes significantly decreased starting in 2008, more than 70% of Korean adolescents can still purchase cigarettes. Enforcement of the Juvenile Protection Act must be strengthened in order to prevent cigarette use among adolescents.

**Electronic supplementary material:**

The online version of this article (10.1186/s12889-018-6151-9) contains supplementary material, which is available to authorized users.

## Background

The prevalence of current (past 30 days) adolescent cigarette smoking in Korea was 9.6% among adolescent males and 2.7% among adolescent females in 2016 [1]. Although these percentages are the lowest since the past decade, the initiation age for daily cigarette smoking among Korean adolescents has been decreasing since 2005. In 2005, the initiation age for daily cigarette smoking was 14.1 years among adolescent males and females; however, it decreased to 13.7 years among adolescent males and 13.5 years among adolescent females in 2015 [2]. These initiation ages are much lower in comparison to 17.6 years in Europe and 17.7 years in New Zealand [3, 4]. Furthermore, the percentages of adult smokers who initiated smoking at ≤18 years were 45.6% in men and 33.4% in women in 2012 [5]. The decrease in the initiation age for daily cigarette smoking emphasizes the need for actions to reduce youth access to tobacco products. Early smoking initiation is especially detrimental to health because it increases risks of experiencing smoking-related morbidities and all-cause mortality independent of demographic characteristics and subsequent smoking intensity [6]. Furthermore, nicotine addiction increases when smoking begins early [7].

Article 16 of the Framework Convention on Tobacco Control prohibits the sale of tobacco products to minors [8]. It is well-known that limiting the age for cigarette purchase reduces the prevalence of adolescent smoking if it is enforced [9–11]. In Korea, tobacco is regarded as a harmful substance for juvenile use, and no one is allowed to sell, rent, or distribute tobacco products to adolescents, according to the Juvenile Protection Act [12]. Anyone who does not comply is punished by imprisonment with prison labor for not more than two years or by a fine not exceeding 20 million won ($17,652). Despite the laws to protect adolescents from smoking, the currently reported smoking rates indicate that somehow adolescents have access to tobacco products. This implies that the current Juvenile Protection Act is not fully enforced. Point of sale advertisement and the display of cigarettes influence adolescent smoking, and these are prevalent in Korea. The Tobacco Business Act permits only signs, stickers, and posters in tobacco shops; however, 100% of convenient stores had tobacco advertisements, and each convenient store near schools had approximately 25 tobacco advertisements [13]. Although 20 countries have implemented point-of-sale display bans as of 2016 and point-of-sale bans reduce smoking prevalence, advertisement within the retailer’s business place is permitted in Korea [14].

Adolescents obtain cigarettes via either social or commercial sources. It is important to recognize the proportions and characteristics of adolescents who could purchase cigarettes because different approaches would be necessary to prevent cigarette acquisition according to its sources. For instance, cigarette purchase can be controlled through law enforcement interventions with tobacco product sellers, whereas controlling the social sources of cigarettes, such as borrowing or stealing from other youths or adults, requires a more individualized approach. Furthermore, the characteristics of adolescents who purchase cigarettes are different from those of adolescents who obtain cigarettes from other sources. Previous studies have reported that older age, being a male, weekly expenditure, amount of daily cigarette smoking, and any instance of binge drinking were associated with cigarette purchase from a store [15, 16]. Most studies on the ease of cigarette purchase among adolescents were performed in Western countries, and to our knowledge, the issue has not yet been reported in Korea. Furthermore, data evaluating the effectiveness of the current laws limiting adolescents’ cigarette access in Korea is limited.

Therefore, in this study, we investigated the trends in the ease of cigarette purchase, defined as the proportion of adolescents who were able to purchase cigarettes from among those who ever tried to purchase cigarettes in the past 30 days. We further evaluated the associated characteristics of the ease of cigarette purchase.

## Methods

### Study participants

This study was based on data collected in the 1st to 12th Korea Youth Risk Behavior Web-based Survey (KYRBWS) (2005–2016), a nationwide representative cross-sectional survey of Korean middle and high school students aged 12 to 18 years [17]. The KYRBWS is an anonymous, self-administered online survey conducted by the Korea Centers for Disease Control and Prevention to identify the health behaviors of Korean adolescents using a clustered, stratified, multistage probability sampling method. Additional information about the study methodology and design has been reported previously [18]. From 2005 to 2016, the survey covered a total of 800 schools, including 400 middle schools and 400 high schools, with a total of 855,763 respondents (58,717 to 75,643 in each year). The response rate ranged from 89.7% in 2005 to 97.7% in 2010. From among these participants, we analyzed 95,491 adolescents who had tried to purchase cigarettes in the past 30 days. The KYRBWS was approved by the institutional review board of the Korea Centers for Disease Control and Prevention (2014-06EXP-02-P-A), and all participants and their parents or legal guardians provided informed consent to participate in the KYRBWS.

### Measures

#### Sociodemographic characteristics

Demographic characteristics including sex, grade, geographic area, and self-reported socioeconomic status (SES) were collected. In the Korean school system, both middle and high schools consist of three grades. Geographic areas were categorized into the following three groups according to the sampling design of the KYRBWS, metropolitan cities, small and medium sized cities, and rural areas, such as district or county. Self-reported SES was collected as high, middle-high, middle, low-middle, and low, and we categorized middle-high, middle, and low-middle SES as middle SES; thus, the final categories for SES were high, middle, and low.

#### Ease of cigarette purchase

The ease of cigarette purchase among adolescents was evaluated through the question, “How was it when you tried to purchase cigarettes from stores in the past 30 days?” The response options were “I did not try to purchase cigarettes in the past 30 days,” “It was impossible to purchase cigarettes,” “I was able to purchase cigarettes with a lot of effort,” “I was able to purchase cigarettes with little effort,” and “I easily purchased cigarettes without any effort.” Since our study population only included those who had tried to purchase cigarettes in the past 30 days, participants who answered “I did not try to purchase cigarettes in the past 30 days” were excluded. The ease of cigarette purchase was categorized into “Yes” when participants answered “I was able to purchase cigarettes with a lot of effort,” “I was able to purchase cigarettes with little effort,” or “I easily purchased cigarettes without any effort” and “No” when participants answered “It was impossible to purchase cigarettes.”

#### Other variables

The average amount of cigarette smoking in one day was categorized as < 1 cigarette, 1 cigarette, 2–5 cigarettes, 6–9 cigarettes, 10–19 cigarettes, and ≥ 20 cigarettes. Quit attempts in the past year, current smokers among family members, and close friends who smoke were surveyed as binary outcomes, and the participants answered either “Yes” or “No” to the questions. Problematic drinking in the past year was defined as participants’ experience of one of the following: drinking alcohol to relieve stress or get along with others; drinking alone; family members or friends asking to reduce drinking; driving a motorcycle or a bicycle after drinking or riding in a car, a motorcycle, or a bicycle driven by a person who drank; alcohol-induced blackouts; or fighting with others after drinking. The amount of weekly pocket money was categorized into < 30,000 won ($26.88), 30,000–59,999 won ($26.88–$53.75), 60,000–89,999 won ($53.75–$80.63), 90,000–119,999 won ($80.63–$107.51), 120,000–149,999 won ($107.51–$134.38), and ≥ 150,000 won ($134.38). The participants were asked about the most common ways of cigarette acquirement in the past 30 days and chose one of the following: acquired from one’s own or a friend’s house, bought from a store, borrowed from friends, borrowed from adults, or picked up from the streets.

### Statistical analyses

All analyses were performed after accounting for the sample weights and complex sample design of the surveys [18]. The prevalence of the ease of cigarette purchase from 2005 to 2016 was calculated, and odds ratios (ORs) and 95% confidence intervals (CIs) of the ease of cigarette purchase each year were compared with those of 2005 using logistic regression models. The Chi-squared test was used to compare the categorical variables, and data were presented as unweighted numbers and weighted percentages. The characteristics associated with the ease of cigarette purchase were evaluated with multivariate logistic regression analyses after adjusting for potential confounders. Since two variables (current smokers among family members and current smokers among close friends) used in the analysis were only available in the 10th to 12th KYRBWS, multivariate logistic regression analyses were performed using data from 2014 to 2016. All analyses were conducted with IBM SPSS Statistics for Windows, Version 23.0 (IBM Corp., Armonk, NY, USA), and two-tailed *p*-values < 0.05 were considered statistically significant.

## Results

### Demographic and smoking-related characteristics of the study participants

Among 855,763 respondents, a total of 95,491 participants who had tried to purchase cigarettes in the past 30 days were included in the study. Table 1 lists general characteristics of the study participants and the Chi-squared test results between ease of cigarette purchase and characteristics of the study participants. More than 80% of adolescent males and females were able to purchase cigarettes. The proportions of students who were able to purchase cigarettes easily increased with grade and average amount of cigarette smoking in a day. More than 80% of the participants were able to purchase cigarettes regardless of geographic area; however, those in metropolitan cities had more opportunities to purchase cigarettes. The proportion of students who were able to purchase cigarettes was significantly greater among those with low SES, who had problematic drinking in the past year, who did not have any quit attempts in the past year, who had current smokers among family members, and who had current smokers among close friends (*p* < 0.05). The proportions of students who were able to purchase cigarettes generally increased as the amount of weekly pocket money increased.

**Table 1:** Basic characteristics of the study participants in the KYRBWS 2005–2016 (*N* = 95,491)

	Ease of cigarette purchase				
	Yes (*n* = 76,860)		No (*n* = 18,631)		*p*-value^a^
	N	%	N	%	
Sex					
Adolescent males	54,496	80.8	13,205	19.2	0.691
Adolescent females	22,364	80.7	5426	19.3	
Grade					
Middle 1	2967	51.3	2889	48.7	< 0.001
Middle 2	6338	65.1	3420	34.9	
Middle 3	10,372	74.6	3534	25.4	
High 1	16,758	83.3	3409	16.7	
High 2	20,040	86.6	3070	13.4	
High 3	20,385	90.0	2309	10.0	
Area					
Rural	10,304	80.3	2567	19.7	
Urban (small and medium sized cities)	30,841	80.2	7767	19.8	0.001
Urban (metropolitan cities)	35,715	81.4	8297	18.6	
Self-reported socioeconomic status					
High	6351	79.7	1720	20.3	
Middle	61,982	80.6	15,143	19.4	< 0.001
Low	8527	83.2	1768	16.8	
Problematic drinking in the past year					
No	25,331	68.3	12,135	31.7	< 0.001
Yes	51,529	89.0	6496	11.0	
Average amount of cigarette smoking per day					
< 1 cigarette	4982	73.5	1835	26.5	< 0.001
1 cigarette	3393	76.4	1080	23.6	
2–5 cigarettes	18,417	85.2	3109	14.8	
6–9 cigarettes	16,077	91.3	1530	8.7	
10–19 cigarettes	11,958	94.1	794	5.9	
≥ 20 cigarettes	5524	94.4	336	5.6	
Quit attempts in the past year					
No	8258	88.1	1184	11.9	0.026
Yes	22,815	87.1	3492	12.9	
Current smokers among family members					
No	3856	74.2	1426	25.8	< 0.001
Yes	7791	76.9	2440	23.1	
Current smokers among close friends					
No	654	33.3	1371	66.7	< 0.001
Yes	11,591	81.8	2652	18.2	
Pocket money (won/week)^b^					
< 30,000	32,247	74.8	10,902	25.2	< 0.001
30,000-59,999	18,589	83.8	3671	16.2	
60,000-89,999	4921	87.5	754	12.5	
90,000-119,999	3264	88.0	475	12.0	
120,000-149,999	903	89.6	100	10.4	
≥ 150,000	5187	87.5	740	12.5	

*KYRBWS*: Korean Youth Risk Behavior Web-Based Survey

^a^P-values were calculated by Chi-squared test

^b^Currency exchange for U.S. dollar: 30,000 won = $26.88; 60,000 won = $53.75; 90,000 won = $80.63; 120,000 won = $107.51; 150,000 won = $134.38

### Ways of cigarette acquisition

Table 2 lists the most common ways of cigarette acquisition among the study participants. In the order by frequency, these methods were in the order of purchasing (69.8%), borrowing from friends (19.5%), obtaining from one’s house (5.2%), borrowing from adults (2.8%) and picking up from the streets (2.8%). Adolescents who purchased cigarettes were able to obtain cigarettes more easily (96.5%) than those who acquired cigarettes through other methods.

**Table 2:** Ways of cigarette acquirement according to the ease of cigarette purchase, KYRBWS 2005–2016 (N = 95,491)

	Ease of cigarette purchase				
	Yes (*n* = 76,860)		No (*n* = 18,631)		*p*-value^a^
	N	%	N	%	
Acquired from one’s own or a friend’s house	2973	5.2	1457	17.1	
Bought from stores	39,410	69.8	1412	17.7	
Borrowed from friends	11,044	19.5	4170	51.8	< 0.001
Borrowed from adults	1465	2.8	532	6.9	
Picked up from streets	1466	2.8	534	6.5	

^a^P-values were calculated by Chi-squared test

### Trends in the ease of cigarette purchase from 2005 to 2016

Table 3 and Additional file 1: Figure S1 illustrate the trends in the ease of cigarette purchase from 2005 to 2016. In general, the ease of cigarette purchase decreased from 2005 to 2016 (p for trend < 0.001), and it continuously decreased since 2008 (OR 0.71, 95% CI 0.64–0.80). There were two points at which the ease of cigarette purchase significantly decreased compared to the previous year. It decreased from 80.9% (OR 0.65, 95% CI 0.58–0.72) in 2012 to 76.5% (OR 0.47, 95% CI 0.42–0.53) in 2013. It decreased once again from 79.3% (OR 0.50, 95% CI 0.44–0.57) in 2015 to 71.4% (OR 0.32, 95% CI 0.28–0.36) in 2016. The non-overlapping CIs between the two consecutive years indicate that there was a significant decrease in the ease of cigarette purchase.

**Table 3:** Trends in the ease of cigarette purchase, 2005–2016

Year	Ease of cigarette purchase					
	Yes	No	Model 1^a^		Model 2^b^	
	N (%)	N (%)	OR	95% CI	OR	95% CI
2005	4851 (83.9%)	860 (16.1%)	1.00	–	1.00	–
2006	6898 (86.5%)	1059 (13.5%)	1.22	1.08–1.38	1.00	0.88–1.13
2007	9021 (84.1%)	1722 (15.9%)	1.01	0.90–1.14	0.90	0.80–1.01
2008	8657 (81.3%)	2013 (18.7%)	0.83	0.74–0.93	0.71	0.64–0.80
2009	7829 (80.5%)	1982 (19.5%)	0.79	0.70–0.89	0.66	0.59–0.74
2010	7552 (80.9%)	1777 (19.1%)	0.81	0.72–0.92	0.69	0.61–0.77
2011	7415 (81.0%)	1787 (19.0%)	0.82	0.73–0.92	0.68	0.61–0.76
2012	6748 (80.9%)	1617 (19.1%)	0.81	0.72–0.91	0.65	0.58–0.72
2013	5644 (76.5%)	1791 (23.5%)	0.62	0.55–0.70	0.47	0.42–0.53
2014	4957 (76.9%)	1568 (23.1%)	0.64	0.57–0.72	0.46	0.41–0.52
2015	4028 (79.3%)	1074 (20.7%)	0.73	0.65–0.83	0.50	0.44–0.57
2016	3260 (71.4%)	1381 (28.6%)	0.48	0.42–0.54	0.32	0.28–0.36

^a^Model 1: Crude analysis

^b^Model 2: Adjusted for sex, grade, area, and self-reported socioeconomic status

### Association between ease of cigarette purchase and participant characteristics

Table 4 presents the multivariate logistic regression analyses of characteristics associated with the ease of cigarette purchase. After adjusting for year, sex, grade, area, self-perceived SES, problematic drinking in the past year, quit attempts in the past year, current smokers among family members, current smokers among close friends, pocket money, and average amount of daily cigarette smoking, the ORs for ease of cigarette purchase significantly increased with grade and average amount of daily cigarette smoking. Furthermore, the ORs for ease of cigarette purchase significantly increased in those who lived in metropolitan areas compared to the rural dwellers, those who had problematic drinking in the past year, and those who had current smokers among close friends. The OR decreased in those who had current smokers among family members. Regarding weekly pocket money, the OR generally increased in those who received more pocket money.

**Table 4:** Odds Ratios from weighted multivariate logistic regression analysis predicting ease of cigarette purchase among Korean adolescents, KYRBWS 2014–2016 (*N* = 16,268)

	Crude		Multi-adjusted	
	OR	95% CI	OR	95% CI
Year				
2014	1.00	–	1.00	–
2015	1.15	(1.04–1.28)	0.98	(0.84–1.14)
2016	0.75	(0.68–0.83)	0.85	(0.73–1.00)
Sex				
Adolescent males	1.00	–	1.00	–
Adolescent females	0.82	(0.74–0.90)	1.01	(0.86–1.18)
Grade				
Middle 1	1.00	–	1.00	–
Middle 2	2.71	(2.18–3.37)	1.43	(0.96–2.13)
Middle 3	5.14	(4.12–6.42)	2.05	(1.39–3.03)
High 1	8.63	(6.97–10.67)	2.79	(1.93–4.04)
High 2	11.01	(8.92–13.60)	3.08	(2.14–4.44)
High 3	14.23	(11.52–17.58)	3.64	(2.52–5.25)
Area				
Rural	1.00	–	1.00	–
Urban (small and medium sized cities)	1.04	(0.89–1.23)	1.15	(0.93–1.43)
Urban (metropolitan cities)	1.14	(0.97–1.34)	1.32	(1.06–1.64)
Self-reported socioeconomic status				
High	1.00	–	1.00	–
Middle	1.02	(0.90–1.16)	0.98	(0.78–1.23)
Low	1.55	(1.27–1.88)	0.96	(0.71–1.30)
Problematic drinking in the past year				
No	1.00	–	1.00	–
Yes	4.34	(3.97–4.73)	1.55	(1.36–1.78)
Average amount of cigarette smoking per day				
< 1 cigarette	1.00	–	1.00	–
1 cigarette	1.02	(0.80–1.30)	0.98	(0.76–1.28)
2–5 cigarettes	1.87	(1.55–2.26)	1.55	(1.27–1.90)
6–9 cigarettes	3.79	(3.04–4.72)	2.84	(2.24–3.59)
10–19 cigarettes	5.50	(4.27–7.08)	4.29	(3.26–5.65)
≥ 20 cigarettes	6.58	(4.75–9.10)	5.10	(3.56–7.31)
Quit attempts in the past year				
No	1.00	–	1.00	–
Yes	0.85	(0.74–0.98)	0.96	(0.82–1.11)
Current smokers among family members				
No	1.00	–	1.00	–
Yes	1.16	(1.07–1.26)	0.87	(0.76–0.99)
Current smokers among close friends				
No	1.00	–	1.00	–
Yes	9.01	(8.02–10.13)	1.66	(1.22–2.27)
Pocket money (won/week)^a^				
< 30,000	1.00	–	1.00	–
30,000-59,999	1.81	(1.65–1.98)	1.27	(1.10–1.46)
60,000-89,999	2.39	(2.02–2.84)	1.13	(0.88–1.45)
90,000-119,999	2.62	(2.10–3.27)	1.32	(0.97–1.79)
120,000-149,999	3.89	(2.40–6.30)	2.90	(1.19–7.06)
≥ 150,000	3.11	(2.58–3.74)	1.48	(1.09–2.01)

^a^Currency exchange for U.S. dollar: 30,000 won = $26.88; 60,000 won = $53.75; 90,000 won = $80.63; 120,000 won = $107.51; 150,000 won = $134.38

*KYRBWS*: Korean Youth Risk Behavior Web-Based Survey

## Discussion

The proportion of the adolescents who were able to purchase cigarettes was large in Korean adolescents; however, it tended to decrease since 2008 compared to 2005. Furthermore, there was a significant decrease from 2012 to 2013 and from 2015 to 2016. Adolescents were more likely to obtain cigarettes in their attempts as their grades were higher, when they lived in metropolitan cities, when they had problematic drinking in the past year, as the amount of cigarette smoking per day was higher, and when they had current smokers among close friends. They were less likely obtaining cigarettes if there were current smokers among family members. The relationship between ease of cigarette purchase and weekly pocket money was inconsistent; however, adolescents who received more than 120,000 won ($107.51) were more likely to make successful attempts than those who received less than 30,000 won ($26.88).

Although the ease of cigarette purchase is decreasing, more than 70% of adolescents in Korea could obtain cigarettes when they attempted to purchase them. This implies that the law is not properly enforced. Situations are similar in other countries [19–22]. For instance, approximately 90% of adolescents in the United States had “very easy” perceived access to cigarettes, even though the exact percentage of adolescents who were able to purchase cigarettes was not reported [19]. Only 45% of adolescents were asked to show proof of age when attempting to buy cigarettes, according to the study of Filippidis et al. [20]. In Canada, the prevalence of students in grade 9 to 12 who thought “it was easy to get cigarettes” was 95.8% among those who had smoked in the past 30 days [21]. Furthermore, 56.5% of adolescent smokers obtained cigarettes from commercial sources in the European region and 75.1% of smokers aged 13 to 15 years were allowed to buy cigarettes despite being underage, according to the data of the Global Tobacco Surveillance System [22]. The tobacco sales ban for adolescents is important because a decrease in buying tobacco is associated with a decrease in smoking prevalence [23]. Effective enforcement of age-restricted tobacco sales laws has constrained adolescents’ attempts of cigarette purchase and further reduced smoking prevalence [10, 24].

Compared to 2005, the ease of cigarette purchase began to decrease since 2008, and a further decrease was noted from 2012 to 2013 and from 2015 to 2016. There has been a strengthening of many tobacco control policies to reduce tobacco consumption since 2005. Public health centers began to offer smoking cessation clinics, which are open to both adults and minors, and adolescents can visit the clinics without their parents. Smoking in restaurants and bars was banned in 2013; cigarette prices were increased in 2005 and 2015; and pictorial health warnings on cigarette packs were introduced in 2016. Anti-tobacco mass media campaigns have been active since 2015 to change smoking-related social norms to influence the perception and behavior of smokers [25–27]. We do not have information on whether these policies are effective at reducing the ease of cigarette purchase; however, all of these factors may have contributed to changes in social norms and decreased underage cigarette purchases. Furthermore, the Juvenile Protection Act was amended in March 2015. The new regulation indicates that when any person sells a juvenile a harmful substance including tobacco products, he/she should post “We do not sell tobacco to minors” in the relevant business establishment [12]. This change in the Juvenile Protection Act may have made tobacco product sellers cautious and further decreased the percentages of adolescents who were able to purchase cigarettes in 2016. It is less likely that the crackdown on violations of law decreased the ease of cigarette purchase because the number of crackdowns has remained more or less the same since 2006 [28].

Adolescents were more likely to obtain cigarettes when they tried to purchase as their grades and the amount of cigarette smoking per day increased. In general, the prevalence of smoking increases with age among adolescents, and the amount of cigarette smoking increases as their smoking period increases [29]. Therefore, students in higher grades and who have a higher amount of daily cigarette smoking would have more experience in smoking and purchasing cigarettes from stores. Furthermore, it is difficult to identify high school students when they dress like adults. Hence, checking identification cards is essential to minimize the number of adolescents with experience in tobacco product purchase, and checking identification was found to decrease the current smoking of adolescents in many studies [30, 31]. The current Juvenile Protection Act requires those who intend to sell, lend, or distribute tobacco products to another person to verify the person’s age [12]. However, 29.2% of students aged 13 to 15 years were not prevented from buying cigarettes because of their age [32]. Regulatory agencies need to increase surveillance for enforcement of the Juvenile Protection Act.

In terms of tobacco purchase experience, weekly pocket money may be another factor influencing the ease of purchasing cigarettes because students with more money can try to buy cigarettes more often. In our analyses, the relationship between ease of cigarette purchase and weekly pocket money was rather inconsistent; however, adolescents with a larger amount of weekly pocket money obtained cigarettes more easily than those who received the least amount of pocket money. These results are consistent with analyses of the Canadian Youth Smoking Survey [16, 21]. The prevalence of students who thought “It is easy to get cigarettes” increased as weekly spending money increased, and spending more than $100 per week was associated with cigarette purchase from a store. Furthermore, adolescents who bought cigarettes from stores were able to acquire cigarettes easier than those who obtained cigarettes through other methods in our analyses (Table 2), indicating that tobacco purchase experience may increase the likelihood of obtaining cigarettes from a store. It has been reported that number of years since starting smoking and average number of cigarettes per day were associated with adolescents’ behavior of buying their own cigarettes from a store [16]. More experience of smoking would increase the ease of cigarette purchase among adolescents.

In Canada and India, the prevalence of cigarette smoking among adolescents is higher in rural than urban areas [33, 34]. In spite of the greater prevalence of adolescent smoking in rural areas [35, 36], adolescents in metropolitan cities were more successful when trying to purchase cigarettes. This may suggest an insufficient enforcement of the Juvenile Protection Act in metropolitan and big cities.

Adolescents who drink alcohol are more likely to smoke, and smoking is especially common among adolescents treated for alcohol use disorders [37]. In our analysis, adolescents were more likely to make successful attempts at cigarette purchases when they had problematic drinking in the past year. Those with more experience in purchasing illegal products easily obtained cigarettes as well. Besides, more than 60% of the study participants (57,998 out of 95,491) had an experience of problematic drinking in the past year. Previous studies have reported that the experience of binge drinking and its frequency were associated with buying cigarettes [16, 38]. This implies that more than 60% of Korean adolescents are exposed to hazardous environments that may lead to cigarette purchase.

Adolescence is a life stage in which one’s behavior is easily influenced by surrounding environments including family members and friends [39]. Adolescents were more likely to purchase cigarettes successfully when they had current smokers among their close friends because they would mimic their friends’ behavior of purchasing tobacco products. This phenomenon was also reported in the study of O’Loughlin et al. [40]. In contrast, adolescents were less likely to purchase cigarettes successfully if there were current smokers among their family members. It has been reported that adolescents are more likely to obtaining cigarettes from an adult or steal them from others rather than buy cigarettes from a store or obtain them from another adolescent when they have other smokers in the household [41]. Adolescents who have current smokers among family members make fewer attempts to purchase cigarettes; thus, their success rate is lower than that of other adolescents.

There are several laws made especially to protect adolescents from tobacco products in Korea [12]. The Juvenile Protection Act prohibits selling tobacco products to juveniles and requires age verification when selling tobacco products. The National Health Promotion Act requires those who sell tobacco by installing tobacco vending machines to install an adult verification device. In addition, schools, including school buildings, playgrounds, and whole premises, are designated as non-smoking areas. The Tobacco Business Act restricts tobacco sales to wholesalers and retailers, and these retailers cannot sell tobacco to consumers by way of postal sale or electronic transactions in order to prevent youths from obtaining tobacco products by post or delivery service. Nevertheless, our results indicate that more than 70% of Korean adolescents can still purchase cigarettes. This suggests that laws to prevent selling cigarettes to minors are not enforced and behaviors of retailers are not changed. Surveillance of retailers and the process of age verification must be strengthened, and penalization should be carried out when the laws are not followed. Public health professionals should encourage regulatory enforcement agencies to enforce the current laws and educate retailors regarding the problems of adolescent smoking.

There are several limitations in this study. We defined the ease of cigarette purchase as proportions of adolescents who were able to purchase cigarettes from among those who had tried to purchase cigarettes in the past 30 days because this was the definition provided by KYRBWS. If we included participants who were able to purchase cigarettes with little effort or without any effort, the results may have been different. However, including these participants would not provide clear results because “little effort” or “without any effort” is very subjective. Moreover, we pooled the 10th to 12th KYRBWS and performed logistic regression analyses instead of using all survey waves because some covariates were only available from 2014 to 2016. Furthermore, since this study was based on a self-reported survey of adolescents, the responses may have been exaggerated or underreported. In addition, we investigated the associated factors of ease of cigarette purchase using cross-sectional data. Instead of using a self-reported survey, studies inspecting stores by using minors will overcome these limitations.

## Conclusions

In conclusion, the trend in the ease of cigarette purchase is decreasing. It began to decrease from 2008 compared to 2005, and a great decrease was observed from 2012 to 2013 and from 2015 to 2016. However, although the trend is decreasing, more than 70% of Korean adolescents can purchase cigarettes. There has been a strengthening of many tobacco control policies to reduce tobacco consumption since 2005, and the Juvenile Protection Act, the National Health Promotion Act, and the Tobacco Business Act are made to prevent youth smoking. Nevertheless, evidence-based tobacco control policies including monitoring the tobacco selling practice of retailers, education of retailers, and enforcement of the Juvenile Protection Act are needed. Public health professionals should encourage regulatory agencies to enforce the relevant laws and educate retailors regarding the problems of adolescent smoking.

## Additional file

Additional file 1: **Figure S1.** Trends in the ease of cigarette purchase, 2005–2016. (DOC 36 kb)

## Abbreviations

CI: Confidence interval
KYRBWS: Korea Youth Risk Behavior Web-based Survey
OR: Odds ratio
SES: Socioeconomic status
