# Supplementary figures and images for: Trends in the ease of cigarette purchase among Korean adolescents: evidence from the Korea youth risk behavior web-based survey 2005–2016
Source: BMC Public Health. 2018 Nov 7;18:1242. doi: 10.1186/s12889-018-6151-9 (PMC6222989; doi:10.1186/s12889-018-6151-9)

**Figure S1**


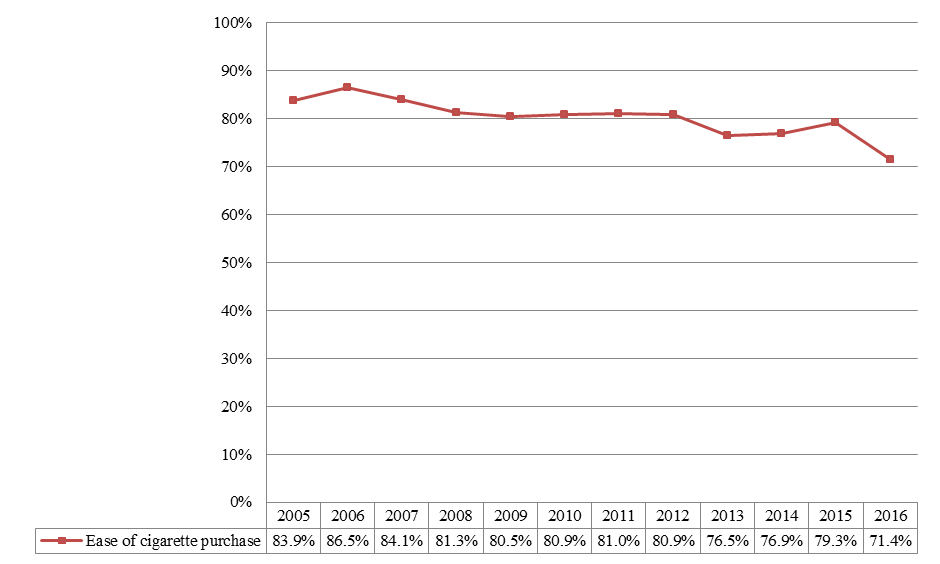

Supplement: Supplementary file 1 — Figure S1. Trends in the ease of cigarette purchase, 2005–2016. (DOC 36 kb) [file 12889_2018_6151_MOESM1_ESM.doc]
